# Roles and Modalities of Ectonucleotidases in Remodeling the Multiple Myeloma Niche

**DOI:** 10.3389/fimmu.2017.00305

**Published:** 2017-03-20

**Authors:** Antonella Chillemi, Valeria Quarona, Luca Antonioli, Davide Ferrari, Alberto L. Horenstein, Fabio Malavasi

**Affiliations:** ^1^Laboratory of Immunogenetics and CeRMS, Department of Medical Sciences, University of Torino, Torino, Italy; ^2^Department of Clinical and Experimental Medicine, University of Pisa, Pisa, Italy; ^3^Department of Life Science and Biotechnology, University of Ferrara, Ferrara, Italy

**Keywords:** ectoenzymes, adenosine, multiple myeloma therapy, microvesicles, CD38

## Abstract

Ectoenzymes are cell surface molecules, which represent functional bridges between the environment and the cytoplasm. One set of ectoenzymes—CD39, CD38, CD203a, and CD73—leads to the generation of adenosine (ADO) by metabolizing ATP and NAD^+^. While ADO is known to control inflammation and suppress immune responses, other aspects of ADO function are still obscure, mainly due to its short half-life in biological fluids. Human multiple myeloma (MM) grows in the closed system of the bone marrow (BM) niche representing an ideal setting for studying ectoenzymes and their products. Another source of information on ectoenzyme function may derive from *in vivo* results of anti-CD38 antibody therapy in MM. Current results, obtained from *in vitro* models and from preliminary *in vivo* findings, indicate that ectoenzymes produce ADO locally in the BM niche. Furthermore, MM cells release microvesicles (MV), which thanks to their molecular cargo and surface ectoenzymes may function as particulate communicators outside of the niche. During anti-CD38 antibody therapy, the MV carry therapeutic IgG, determining that the prevalent orientation of MV will be toward cells and tissues expressing receptors for the IgG Fc domain. The resulting picture is one where MM adopts an immune escape strategy based on reshaping the environmental niche. This adaptation is followed by actions of MV that are exerted in biological fluids and circulating immune cells. By coating FcRs^+^ cells, MV modify pericellular spaces, reproducing the metabolic halo generated by ectoenzymes within closed systems.

## Introduction

The present perspective aims to highlight the contribution of adenosinergic ectoenzymes to the metabolic pathways that contribute to tumor growth and active immune evasion. Part of the knowledge in this field has been inferred indirectly from recent findings obtained within the context of immune tolerance. Supporting evidence has also been from (i) *in vivo* studies on organ transplantation, (ii) the identification of regulatory cell subsets, and (iii) the tolerogenic substances and their control mechanisms. Additional and somewhat unexpected evidence came to light with the introduction of innovative therapeutic drugs. The use of immunomodulators and monoclonal antibodies in cancer therapy revealed previously unknown facets of normal and tumor biology. For example, drug resistance was first observed when it became apparent that cancer cells could actively eliminate most of the toxic drugs used in therapy. Another important result was the identification of cell surface molecules able to drive the production of tolerogenic substances (e.g., adenosine, ADO) and of establishing a tolerogenic environment (e.g., in the bone marrow, BM).

Here, we will focus our attention on a panel of enzymes localized on the cell surface (hence, ectoenzymes) that are involved in the metabolism of ATP and NAD^+^. It is known that evolutionary pressure led to the selection of ATP and Ca^2+^ as an inseparable tandem occurring as a leitmotif in the control of life, death, and cellular signaling. Schematically, the original function of ATP was to provide energy within the cell, while Ca^2+^ served as a key second messenger for extracellular signaling *via* purinergic P_2_ receptors (1).

The main acceptor of extracellular ATP is the ectoenzyme CD39 (also known as ectonucleoside triphosphate diphosphohydrolase 1), which metabolizes the substrate into AMP, subsequently taken up by CD73 (also known as ecto-5′-nucleotidase) and transformed into ADO. This nucleoside exerts modulatory effects on immune and cancer cells by binding P_1_ receptors (2). The function of ADO in inflammation has been the subject of extensive reviews (3).

Over their long course of the history of life, the processes of generating energy and cell signals have evolved to include the glycolytic proton (H^+^) acceptor NAD^+^. The main acceptor of NAD^+^ in the extracellular space is CD38 and CD157 (BST1) both are ADP-ribosyl cyclases/cyclic ADP-ribose (ADPR) hydrolases, although with different catalytic characteristics (4). By consuming NAD^+^, CD38 leads to the generation of ADPR, as well as of a small amount of cyclic ADPR (cADPR), a Ca^2+^-mobilizing second messenger (5). Although cyclase is only a fraction (1–3%) of the enzymatic output of CD38, cADPR functions as a Ca^2+^-modulating agent in multiple cell types (6). In the presence of CD203a (also known as ectonucleotide pyrophosphatase/phosphodiesterase-1 or PC-1), NAD^+^ and ADPR can also serve as additional sources from which to generate AMP (7).

The networks involved in the metabolism of ATP and NAD^+^ are complex and heterogeneous, encompassing a panoply of soluble substrates, on one side, and cell surface receptors operating in different tissues, on the other side. The simplicity of the basic elements originally devoted to the generation of energy seems at odds with the complexity of the receptors involved in the use of their products and is perhaps symptomatic of an antieconomic design flaw that has managed to slip past the pressures of natural selection.

According to current knowledge, ATP and NAD^+^ may be metabolized by distinct ectoenzymatic pathways, one relies upon the presence of CD39 and CD73, the second pathway depends on CD38, CD203a, and CD73. The presence of CD203a enables the two pathways to converge and both may lead to ADO as a final product. It is reasonable to assume that the evolutionary process, which leads to this result, originated from the necessity to adjust the metabolic demand of cells according to the available energy sources and new functions. One might speculate that ADO straddled the two roles of information and energy source as a regulatory mechanism in primitive organisms (8).

Adenosine is considered to have been a central metabolite within the intracellular (e.g., transmethylation) and extracellular (e.g., purinergic) biochemical pathways (9) and also represents a tight link to the energetic reservoir of the cell. Later, ADO developed as a more sophisticated regulatory system allowing fine-tuning of important physiological functions (10).

Such an extensive network of cytoplasmic enzymes, plasma membrane ectoenzymes and receptors, as well as extracellular or intracellular soluble products evolved in such a way as to make the system not operate in an all-or-nothing fashion. On the contrary, this intricate system is highly sensitive and capable of providing just the right biological effects required by a given tissue at a given time. The final results arise from a combination of the intrinsic functional characteristics of the enzymes, their affinity toward their substrates, and the promiscuity derived from a resulting sum of affinities, substrates, and actions. Adding further complexity is the notion that the majority of ectoenzymes are extremely susceptible to both the cytoplasmic and extracellular environment. As a consequence, ectoenzyme surface expression can range from nil to high, and similar variations in expression are also seen in the counterparts of receptors for the enzymatic products. The quantitative modifications are of physiological relevance, implementing negative or positive effects as a function of tissue distribution or functional status.

Some steps of ADO functionality are still waiting to be defined, mainly as a consequence of its active metabolization in biological fluids, such as blood stream, considered as an open system. For these reasons, some hints were derived from diseases taken as models from which to infer conclusions valid in physiology.

## Disease Model: Human Multiple Myeloma (MM)

Human MM offers an ideal setting for dissecting the action of the different ectoenzymes and their products. Indeed, myeloma grows in a BM niche where ectoenzymes are expressed. The niche represents a closed system, where the products of the enzymatic reactions are collected and accessible for medical reasons. Furthermore, one of these ectoenzymes (CD38) has become a therapeutic target of *in vivo* antibody therapy (11).

**Figure 1:**
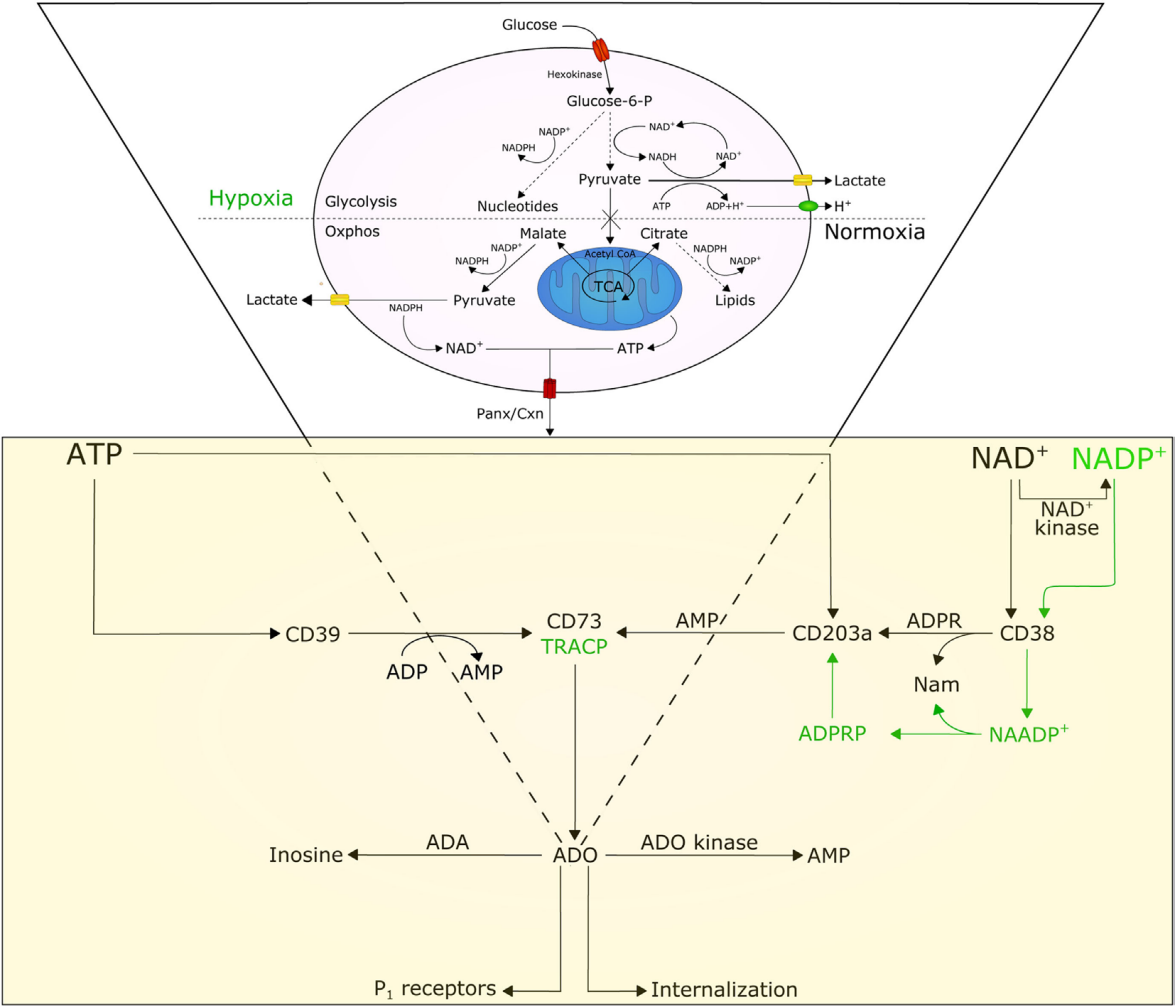
**Model illustrating the involvement and interplay of ATP, NAD^+^, and adenosine (ADO) in normoxic and hypoxic conditions**. Normal cells are characterized by a basal rate of glucose conversion to pyruvate, generating 30 ATPs by mitochondrial oxidative phosphorylation (oxphos). Tumor cells utilize glycolysis instead of oxphos; most of the pyruvate is catalyzed by lactate dehydrogenase A (LDH-A) to lactate producing (i) cytoplasmic NAD^+^, (ii) 2 ATPs, and (iii) protons (H^+^). The efflux of lactate and H^+^ induces acidosis in the tumor microenvironment, which in turn moderates glycolytic metabolism by hexokinase inhibition. Cytoplasmic NAD^+^ and ATP, secreted across Panx/Cxn channels or after cell lysis, are metabolized to ADO in the extracellular space. For details see text.

A few premises are necessary to understand how the BM niche and the cytoplasmic conditions of myeloma cells may influence the functions of ectoenzymes. Tumor growth demands energy; as a consequence, cells may become “addicted” to alternative metabolic pathways, including glucose metabolism for protein, lipid, and nucleotide synthesis (Figure 1) (12). Tumor cells consume glucose at a higher rate than normal cells and secrete most of the glucose-derived carbon as lactate instead of oxidizing it to generate ATP. This phenomenon is known as the Warburg effect. This glycolytic phenotype is an apparently inefficient means of energy production as glycolysis generates two molecules of ATP and two of lactic acid from each molecule of glucose consumed, while oxidative phosphorylation (oxphos, for short) generates about 30 molecules of ATP from 1 molecule of glucose (8). Such a wasteful form of metabolism for generating ATP seems paradoxical. However, this metabolic shift likely provides advantages for proliferating tumor cells that survive under hypoxic conditions. First, the shift allows tumor cells to use extracellular glucose for ATP production, even though at low efficiency. Second, glucose degradation provides tumor cells with the intermediates needed for various biosynthetic pathways, including ribose for nucleotides, glycerol and citrate for lipids, and non-essential amino acids for proteins. These precursors derive from the tricarboxylic acid cycle, a biosynthetic hub that consumes, rather than produces ATP. The high rate of lactate produced remains a controversial topic in research, because most of the pyruvate needs to be oxidized to enhance ATP production. It is likely that the glycolytic flux increases enzymatic activities, creating the need for a system that prevents the accumulation of pyruvate (13). Indeed, the induction of lactate dehydrogenase A observed in tumor cells rapidly consumes pyruvate (14) and regenerates NAD^+^ while yielding lactate and H^+^ as by-products.

Pertinent to the present perspective is that this heritable metabolic shift adopted by the tumor leads to acidification of the extracellular microenvironment, leading to apoptosis of cells unable to survive in such extreme conditions. Another source of H^+^ in the tumor microenvironment is from the hydrolysis of ATP. Over time, lactate and H^+^ are exported from cancer cells into the interstitial space, where they accumulate because of defective blood perfusion. The result is further acidification of the extracellular tumor microenvironment. Acidosis can thus be considered as a tool selected by tumor cells to bypass in growth normal cells (15).

In the course of evolution, tumors have learnt to exploit weaknesses in the immune system, evading defenses and proliferating at a distance from the original neoplastic site. As in inflammatory lesions, the tumor environment is characterized as a locus of decreased pH (16, 17), which is on average 0.5 U lower than in normal surrounding tissues (16). This is confirmed in several disease models, including MM. MM grows in a niche localized in the BM, where tumor cells are secluded in a physically constrained three-dimensional site surrounded by different cells (where osteoblasts, osteoclasts, stromal cells, and mesenchymal stem cells are dominant). Cells are flowed by plasma fluids, which act as a liquid communicator among the different cell components (9). Plasma and cells inside the BM are aspirated for diagnostic and therapeutic purpose from MM patients. This makes it possible to closely track events that occur within the niche and assess their resulting products.

Studies from basic and clinical science have made it possible to delineate the different paths and processes that favor growth and expansion of myeloma cells. The conclusions of these studies are potentially transferable to other diseases (18). It is generally accepted that MM exploits the capacity of pH to modify the environment and the immune response within the context of the niche. This is confirmed by the observation that low pH in BM plasma is paralleled by (i) phenotypic modifications of the cells surrounding the tumor and (ii) by increased levels of locally produced ADO.

Hypoxia inside MM cells is exploited to reprogram cellular metabolism so that aerobic glycolysis can satisfy the tumor’s additional energy requirement. Hypoxia outside the MM cells helps to modify the nucleotide metabolism, where ATP and NAD^+^ are metabolized by the surrounding cells expressing surface proteins endowed with ectonucleotidase activity. Besides serving as signaling molecules, nucleotides are also rapidly metabolized by specific ectoenzymes into other active nucleotides, and then converted into ADO (19). ADO performs multiple functions, depending on the environment (16). In the immune system, ADO has predominantly suppressive effects and operates through the binding of different types of P_1_ receptors (10). This ultimately leads to a generalized anergic status of T, NK, and dendritic cells, which is considered to be instrumental for myeloma cells immune evasion.

A further effect caused by the increasing local hypoxic conditions is a shift of tumor metabolism from generating ATP to accumulating high levels of NAD^+^ (20). In fact, aerobic glycolysis simultaneously metabolizes glucose to pyruvate, with robust production of NAD^+^ (Figure 1), which is required for cell proliferation (21). This intracellular accumulation of NAD^+^ favors its transmembrane efflux from tumor cells *via* Cnx or Pnx1 hemi channels (22, 23). On one side, this efflux constitutes a unique system that feeds ectonucleotidases (22); on the other, it confers growth advantage to the cells through increased autocrine/paracrine NAD^+^ signaling, also through interactions with the CD38 ectoenzyme on the same membrane. The metabolic halo formed following NAD^+^ efflux may also influence the action of CD38 localized on adjacent non-tumor cells. Thus, micromolar increases in extracellular NAD^+^ concentrations secondary to cell death or lysis may exert local suppressive effects through activation of purinergic P_2_ receptors expressed by immune cells. This results in the induction of cell death in cytotoxic T effectors and expansion of suppressor T lymphocytes (Treg) (24).

A further effect of hypoxia is obtained by working together with selected cytokines to modulate the adenosinergic pathways, privileging an alternative pathway of ADO production (9). Indeed, the above conditions induce expression of the ectoenzymes CD203a and CD73, which are re-expressed or up-modulated by cells involved in NAD^+^ degradation. This was confirmed in the MM niche. Indeed, BM aspirates from MM patients indicated that the canonical adenosinergic CD39/CD73 pathway is flanked by another set of ectoenzymes, which *in vivo* leads to the production of ADO, this time using NAD^+^ as the starting substrate (2). In sequence, extracellular NAD^+^ is metabolized by CD38, CD203a, and CD73, confirming observations obtained *in vitro* in a human T leukemia line (7) and *in vivo* in human NK cells (25).

These ectonucleotidases operate in a discontinuous fashion (meaning that the components of the pathway do not need to be expressed by the same cell), but the metabolic halo around the cells forms a transmission chain of reaction products. A requisite is that the reactions take place in a closed microenvironment, where the tumor and host cells are intermingled located side by side. As a consequence, the metabolic activities in the BM niche promote the local accumulation of ADO, contributing to a local impairment of the immune status (26).

In conclusion, the different cellular components in the niche harbor at least two distinct pathways (CD203a/CD73 and CD38/CD203a/CD73), both potentially leading to the production of ADO. The niche provides the physical proximity necessary for the generation of intermediates and products, while the halo of enzymatic products surrounding juxtaposing cells mechanistically guarantees the functional effects of ADO, whose half-life is extremely short *in vivo*.

Multiple myeloma patients are characterized by a generalized impairment of the immune defenses. It therefore remains to be seen whether the observations seen in the niche may also hold true outside of a closed environment.

### Beyond the Halo

Similar to other tumors, MM spontaneously releases microvesicles (MV) as part of a normal membrane dynamics (27). MV can originate from either the cell membrane or from the cytoplasm (28). The phenotype of membrane-derived MV is highly reminiscent of the cells from which they originated. Surprisingly, MV derived from MM cells strongly express ectoenzymes, such as CD38, CD39, CD73, and CD203a. A possible interpretation is that CD38 is expressed in a membrane domain with active or passive grouping of specialized enzymatic molecules. In any case, the upshot is that MV from MM are equipped with enzymatic apparata potentially capable of metabolizing both ATP and NAD^+^ to produce ADO. When derived from the cell membrane, MV can adhere and fuse with adjacent cells, which promote their egression from the MM niche and migration through tissues from where they eventually reach the blood circulation. Naturally occurring MV may adhere to cells, simply guided by their surface and adhesion molecules. This situation changes after treatment with therapeutic antibodies, when MV are coated with IgG. The consequence is that MV are driven as their preferred destination toward cells and tissues expressing FcRs with different affinities. MV adherence to and accumulation around cells delineates a pericellular space, one side formed by MV and their halo and the other by the membrane of the normal cells. The pericellular space reproduces what is seen in closed environment, with synthesis and use of short-lived products, as in this case with ADO (29).

An even more challenging question is what happens when IgG-coated MV enters a cell. These MV have a cargo of proteins, lipids, nucleotides, and nucleic acids, whose functional effects are under scrutiny. Preliminary evidence indicates that the most up-modulated genes concern immune response, regulation of cell death and apoptosis, chemotaxis, and the inflammatory response.

The identification of molecules that act as enzymes on the cell surface but whose final products are destined for use within the cytoplasm suggests that we urgently need to rethink our understanding of extracellular metabolism.

It is generally accepted that cells surrounded by a halo of functional products can be sensed by adjacent cells, provided that the latter are equipped with the proper receptors. In the case of ADO, a marked functional difference is its limited half-life, making it act more like a neurotransmitter, in that it is operative only within a short distance from the production site. An improvement may come when ADO is produced in the context of a tissue or closed system, which facilitates the interaction among biological fluids and cells.

**Figure 2:**
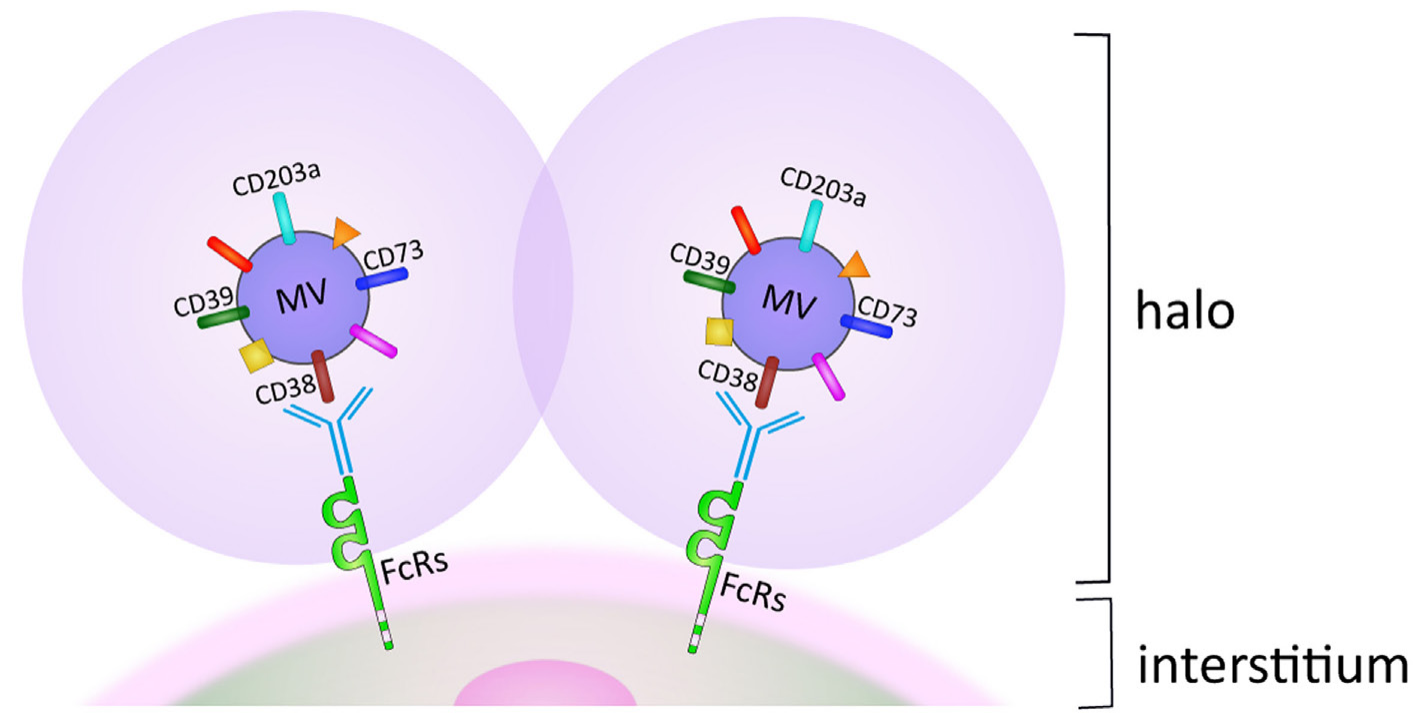
**Halo generated by microvesicles (MV) accumulation on membranes of FcR^+^ cell subsets in biological fluids**. For details, see text.

Is it possible that ADO functions the same way as hormones do and functions at sites distant from the point of origin? If so, it may be that they rely on MV released from the tumor cell membranes. The phenotype of MV is characterized by clusters of ectoenzymes bound for biological fluids and blood located at a distance from their origin. This situation significantly changes in case of *in vivo* therapy with therapeutic antibodies, when they target ectoenzymes (e.g., CD38 and more recently CD73) (30, 31). In this case, the presence of a therapeutic antibody on their surface restricts their final destination to all the body cell populations expressing FcRs (Figure 2) (32).

## Author Contributions

AC and VQ contributed to the experimental part behind this perspective, and edited both text and graphical part. LA and DF revised the work in a critical and constructive way. AH and FM contributed to the conception and the design of the work. All the authors approved the final version of the manuscript and agreed to be accountable for all aspects of the work.

## Conflict of Interest Statement

The authors declare that the research was conducted in the absence of any commercial or financial relationships that could be construed as a potential conflict of interest.
